# Influence of Cardiovascular Risk Factors and Metabolic Syndrome on Epicardial Adipose Tissue Thickness in Rural Spanish Children and Adolescents

**DOI:** 10.3390/nu16193321

**Published:** 2024-09-30

**Authors:** Isabel María Blancas Sánchez, Cristhian H. Aristizábal-Duque, Juan Fernández Cabeza, Manuel Vaquero Álvarez, Pilar Aparicio-Martínez, Manuel Vaquero Abellán, Martín Ruíz Ortiz, María Dolores Mesa Rubio, Francisco Javier Fonseca del Pozo

**Affiliations:** 1 Centro de Salud de Occidente Azahara, Street Campo, 14005 Córdoba, Spain; isabelm.blancas.sspa@juantadeandalucia.es; 2 Grupo Investigación GC09 Nutrigenomics, Metabolic Syndrome, Instituto Maimónides de Investigación Biomédica de Córdoba (IMIBIC), Hospital Universitario Reina Sofía, 14071 Córdoba, Spain; h02vaalm@uco.es; 3 Cardiology Department, Reina Sofia’s University Hospital, Av. Menéndez Pidal, 14004 Córdoba, Spain; chad2155@yahoo.com (C.H.A.-D.); juanfca988@gmail.com (J.F.C.); maruor@gmail.com (M.R.O.); loladoctora@hotmail.com (M.D.M.R.); 4 Grupo Investigación GE10 Clinical and Epidemiological Research in Primary Care, Instituto Maimónides de Investigación Biomédica de Córdoba (IMIBIC), Hospital Universitario Reina Sofía, 14071 Córdoba, Spain; n32apmap@uco.es (P.A.-M.); fjfonsecapozo@yahoo.es (F.J.F.d.P.); 5 Departamento de Enfermería, Farmacología y Fisioterapia, Campus de Menéndez Pidal, Universidad de Córdoba, 14071 Córdoba, Spain; 6 Grupo Investigación GC15 Cardiovascular Diseases, Instituto Maimónides de Investigación Biomédica de Córdoba (IMIBIC), Centro de Investigación Biomédica en Red de Enfermedades Cardiovasculares (CIBER-CV), Hospital Universitario Reina Sofía, 14071 Córdoba, Spain; 7 Faculty of Health Science, Universidad Internacional Isabel I de Castilla, Campus Fernán González, 09003 Burgos, Spain; 8 Distrito Sanitario Córdoba Guadalquivir, Noroeste, 14011 Córdoba, Spain

**Keywords:** epicardial fat, adolescents, cardiovascular risk factors, children, metabolic syndrome

## Abstract

The presence of visceral adipose tissue implies a higher risk of cardiovascular diseases than subcutaneous adiposity, the most dangerous heart fat. Epicardial adipose tissue (EAT) could have great potential as a detection indicator of cardiovascular diseases, although it has seldom been studied in Spanish children. Objective: The objective of the current research was to describe the values of EAT in a Spanish pediatric population and to investigate the associations between EAT and anthropometric measures, blood pressure, lipid and glucose profiles, and metabolic syndrome. Method: An analytical cross-sectional study of elementary and high school students (aged 6 to 17) measured anthropometrics, blood pressure, lipid and glycemic profiles, and echocardiographic fat thickness. The analysis was based on regression and discriminant analysis. Results: The results of this study (N = 227) showed that the body mass index (BMI) was 20.29 ± 4.54, with an overweight set of 49.77%, and the percentage for metabolic syndrome was 5.3%. EAT was linked to being male, BMI percentile, waist circumference, waist-to-height ratio (*p* < 0.001), hypertension, higher low-density lipoprotein (LDL) levels, and metabolic syndrome (*p* < 0.05). Conclusions: This paper argues that in children with higher elevated EAT thickness values, this correlates with cardiovascular risk factors including high blood pressure, elevated LDL levels, and metabolic syndrome.

## 1. Introduction

Adiposity and fat distribution within the human body are critical determinants of the pathogenesis of cardiovascular diseases (CVD) [1]. The accumulation of excess adiposity, particularly visceral fat, has been strongly associated with metabolic disorders such as insulin resistance, dyslipidemia, and hypertension, which collectively contribute to the development of atherosclerosis [2]. These metabolic disorders and CVD are significant public health concerns as they serve as primary risk factors for a range of severe conditions, including diabetes, stroke, cancer [3], and neurodegenerative diseases like Alzheimer’s [4]. The impact of these risk factors is particularly pronounced in adulthood, where the long-term consequences of early metabolic imbalances manifest as chronic illnesses that substantially reduce the quality of life and increase mortality rates [5].

Visceral adipose tissue (VAT) poses a greater health risk than subcutaneous fat, with the severity of this risk varying, depending on the specific organ involved. Among the different types of organ fat, cardiac fat, particularly around the heart, is considered the most dangerous [6]. In fact, an increase in VAT leads to additional fat deposits around the heart, altering the composition of both epicardial and pericardial adipose tissue [3]. Epicardial adipose tissue (EAT) is defined as “the adipose tissue between the myocardium and visceral pericardium, and pericardial fat is the adipose tissue surrounding the parietal pericardium”. EAT has significant potential as a detection indicator since it has several physiological functions, from lipid storage and acting as a mechanical safeguard to the nervous system [7]. Previous studies on adults have established a link between pericardial adipose tissue (PAT) or EAT and an increase in the left ventricle mass and index, carotid intima-media thickness, lipid profile, fasting glucose, and higher blood pressure [7].

Most studies have aimed to detect EAT increases using measures such as waist circumference or the waist-to-hip ratio. More costly imaging techniques, like magnetic resonance imaging (MRI), have also been employed for this purpose [8,9]. However, in recent years, there has been an increase in the use of other imaging detection techniques, mainly echocardiography, based on low accessibility [10]. In this sense, diverse research has associated PAT, and, in some cases, EAT with various health risks and diseases, such as a high body mass index (BMI), high blood pressure or hypertension, metabolic syndrome, and CVD. However, research examining these associations in children and adolescents remains limited, with EAT measurement rarely being included in such studies, despite its significant potential to predict health outcomes [10,11,12]. Moreover, only one study analyzed the impact of diverse factors on the EAT status of children, indicating that children and adolescents from rural areas have lower levels of obesity and EAT values [13].

Early detection is crucial to prevent significant modification to the LVM, metabolic disorders, and CVD, which have become more prevalent among children and adolescents with the passing of the years [10,11,14]. In this sense, indirect measures to identify cardiac structural changes via PAT or EAT have never been applied in children or adolescents [15]. However, not all the indirect parameters are recommended to measure such changes, such as the BMI, since it does not discriminate between total and central tissue deposits [16]. These previous results indicated that a more precise indirect marker would be the waist circumference, although there is an issue when using the BMI combined with waist circumference [17]. Also, there is an increasing tendency to measure the adipocyte tissue of the heart directly via ultrasound, i.e., echocardiography, based on its capacity for detecting CVD and the metabolic changes related to a complete diagnosis in children who are overweight and especially those with obesity [18].

Additionally, it seems that the early detection of CVD in children who are significantly overweight and obese through a combination of anthropometric measurements and echocardiographic study is more precise [19]. Early detection in children is essential for preventing cardiac problems later in their adult years [14] or preventing other health issues such as diabetes or hypothyroidism during adolescence [20]. Nevertheless, in the case of Spain, which exhibits a concerning increase in metabolic disorders, CVD, and overweight or obesity in children or adolescents in rural and urban areas [21,22], there is a lack of studies focused on anthropometric measures, lipidic and glycemic profiles, blood pressure, and PAT, much less on EAT [10,11]. The previous research published in this field that focused on children was carried out in the Netherlands, using PAT, showing significant associations between these different variables, although in urban areas [10]. Understanding the relationship between fat distribution, particularly epicardial adipose tissue, and these diseases is essential for developing targeted prevention and intervention strategies to mitigate the long-term health risks [23]. Therefore, the current study presents unprecedented research in Spain using the EAT, testing its association with different measures for early detection in a rural area of the south.

Based on this finding, the objective of the current research was to describe the values of EAT in a Spanish pediatric population from a rural town and to investigate the associations between EAT and anthropometric measures, blood pressure, lipid and glucose profiles, and metabolic syndrome.

## 2. Materials and Methods

### 2.1. Design and Sample

This descriptive cross-sectional study focused on elementary and high school students aged 6 to 17. The sample was recruited from a small to medium-sized town with around 3000 inhabitants. The sample was recruited to participate in person from March 2018 to February 2019.

The inclusion criteria of participants were their age within a range, obtaining the parents’ or legal guardians’ acceptance at the time of starting the study, and the signed informed consent of the parents or legal guardians, the student him- or herself, and the principal researcher. The exclusion criteria were children under 6 years of age and adolescents over 17, those who were discarded when the study began or who had yet to sign the informed consent form, cardiac pathology, and missing data such as height. The sample selection was based on non-probability convenience sampling, which was based on accessibility. The initial sample size required the participation of 280 subjects, but only 254 subjects fulfilled the criteria. The final sample comprised 227 participants who agreed to undergo the ultrasound procedure (93.7%).

### 2.2. Measures and Instruments

#### 2.2.1. Anthropometric Measurements

Anthropometric variables were measured following the International Standards for Anthropometric Assessment (ISAK) [24] recommendations. Weight was measured in kg with an Omron BF-511 impedance meter (Kyoto, Japan), and height (cm) was measured using a professional portable height rod that was approved and calibrated, a portable model Seca 213^®^ (Hamburg, Germany).

Weight, percentage of fat mass, lean mass, and total body water composition data were obtained with a model Omron BF-511 impedance meter, validated for research studies; the students were dressed in light clothing and were barefoot. The body composition was obtained in a fasting condition without previous exercise. Each schoolchild was classified according to their degree of obesity using the Melo Salor [25] tables, based on the standard deviations (SD) recommended by the World Health Organization regarding weight, height, age, and gender: overweight (+1 SD), obesity (+2 SD), and morbid obesity (+3 SD).

A portable height Seca 213^®^ rod was used. Regarding waist circumference (WC), an inextensible measuring tape was used, passing it through the imaginary line that runs parallel to the ground and through the midpoint between the lower edge of the last rib and the iliac crest; two measurements were made at the end of a normal exhalation, and the measurement was expressed in centimeters.

The fat percentage was calculated using the Faulkner equation [15]. Moreover, the waist-to-height ratio (WHtR) was calculated, using the WC value divided by height (in cm). Finally, the body fat (BF%) percentage and fat-free (FF%) mass were calculated to determine the percentage of fat in the children.

#### 2.2.2. Blood Pressure

Blood pressure (BP) measurement was performed with an automatic monitor validated for research studies, the Omron M6 comfort^®^ model, with blood pressure cuffs suitable for the patient’s arm circumference. BP was measured following the recommendations of the European Society for Arterial Hypertension in children and adolescents [16]. HBP was defined as SBP or DBP levels ≥ the 95th percentile (P95) for a given age, sex, and height percentile. It is necessary to record at least three successive determinations above the P95. Likewise, we considered pre-HTN levels of SBP or DBP ≥ the 90th percentile (P90) but lower than P95. The Spanish Pediatric Association tables were used to classify the study subjects as hypertensive. As hypertension was detected using the oscillometric method, the finding was confirmed using the auscultatory method, and three blood pressure measurements were taken, finally calculating its mean value.

#### 2.2.3. Lipidic and Glycemic Profile

Blood tests were conducted within the first 24 h following sample collection. The results were obtained through spectrophotometric analysis, specifically targeting the blood levels of fasting glucose, HbA1c, total cholesterol, triglycerides, LDL-C, and HDL-C.

#### 2.2.4. Metabolic Status

Metabolic status or MetS is frequently used to define a connection between the pathophysiological modifications related to abdominal obesity, hypertension, dyslipidemia, and glucose. Despite the multiple definitions more commonly used by the IDF [26], Cook et al. [27], Ford et al. [28], and de Ferranti et al. [29], recent research has indicated that the definitions of MetS used by IDF [26], Cook et al. [27] and Ford et al. [28] have similar prevalence [30]. Based on the current sample, the IDF definition was the most adequate, based on a higher level of BP [26].

#### 2.2.5. Echocardiographic Study

Each subject underwent a two-dimensional (2D) transthoracic echocardiogram using the standard technique, with the patients in the left lateral decubitus position. The echocardiogram was performed by a cardiologist, an expert echocardiographer, using an IE33 echocardiographic system and an S5-1 transducer (Philips Medical Systems, Amsterdam, The Netherlands). The thickness of the epicardial fat was measured according to the method validated by Iacobellis et al. [31]. The segmentation of EAT values was established according to the standard values and the confidence interval for no-cardiovascular risks (2.76 ± 0.78) and more than three cardiovascular risks (3.13 ± 0.73), this being identified as the normal expected value and the above interval [32].

The epicardial fat thickness has been identified as the echolucent space between the myocardium’s outer wall and the pericardium’s visceral layer. This thickness was measured perpendicularly over the right ventricle’s free wall at the end of the systole in three cardiac cycles, using a long parasternal or short parasternal view. The measurement was performed on the free wall of the right ventricle for two reasons: (1) this point is anatomically recognized as the one with the most significant thickness of epicardial fat, and (2) the long parasternal and short parasternal axes allow the most accurate measurements of epicardial fat on the right ventricle, with optimal cursor orientation in each view.

#### 2.2.6. Procedure

Participants approved a participant information statement, consent form, and questionnaires, which the Research Ethics Committee then approved.

Before the study and the participants’ inclusion, informative talks were held for parents and students in each educational center. In these meetings, the research’s objectives and methodology were explained, any doubts resolved, and a contact email and telephone number were provided for any subsequent consultation.

The participants were children and adolescents aged between 6 and 17 years, studying in the three centers where the study took place. The anthropometric measurements and cardiography study of each participant were collated in the educational institution by two family doctors with experience. The measurements were taken in each institution over three or four sessions (days) to gather the information, calling in groups of between eight and ten children and representing a session or day per week, usually on Friday.

#### 2.2.7. Statistical Analysis

After obtaining all the data, the SPSS program version 25 was used to analyze each measurement gathered. The absolute and relative frequency were used for the categorical variables. The mean and standard deviation were used for the quantitative calculations, while the qualitative percentages and prevalence were calculated. The normalization tests, Kolmogorov–Smirnov tests with Lilliefors correction, and Q-Q graphs for samples over 50, or the Shapiro–Wilk test for N under 50 were used to compare the goodness-of-fit to an average distribution of data from continuous or discrete quantitative variables [33]. The comparison of two or three independent means was carried out through the correct test (the Mann–Whitney Student test or Student’s *t*-test, and the analysis of variance and Kruskal–Wallis test) according to each variable. For the comparison of percentages, the chi^2^ test was used, with the Yates correction or Fisher’s test when indicated. For the quantitative, bivariate correlation was applied, and Pearson’s correlation coefficient was used. Finally, the associations between EAT thickness and the children’s characteristics and cardiovascular factors were studied through linear multiple regression. The safety and validity indexes were determined to measure the accuracy of the diagnostic tests. Through functions obtained by discriminant analysis, the factors were identified that best classified children with epicardial adiposity.

## 3. Results

The participants (N = 227) were more frequently male, with ages between 11 and 12 (11.32 ± 2.84) years with a range between 7 and 17, and with a confidence interval (CI) of 95% of 10.6 to 11.4. The BMI was set at 20.29 ± 4.54, with the prevalence of obesity and overweight calculated at 49.77%. The results for the lipidic and glycemic profiles were the mean of total cholesterol level (167.35 ± 29.51), triglycerides (73.00 ± 39.79), and HbA1c (5.32 ± 0.03), this being the frequency of metabolic syndrome using the NIM-MetS of 5.3% [30]. The results of the EAT analysis indicated that the mean levels were set at 1.88 ± 0.53 (Table 1). Based on the prevalence of overweight children, the measurements were analyzed according to average weight and overweight. The initial results indicated significant differences between the children and adolescents with average weight and those who were overweight in terms of their body fat percentage (BF%) (*p* < 0.001), free fat percentage (FF%) (*p* < 0.001), waist circumference (WC) (*p* < 0.001), WHtR (*p* < 0.001), systolic blood pressure (*p* < 0.001), cholesterol levels (*p* < 0.01), metabolic syndrome (*p* < 0.05), and epicardial adipose tissue (EAT) thickness (*p* < 0.01) (Table 1).

**Figure 1:**
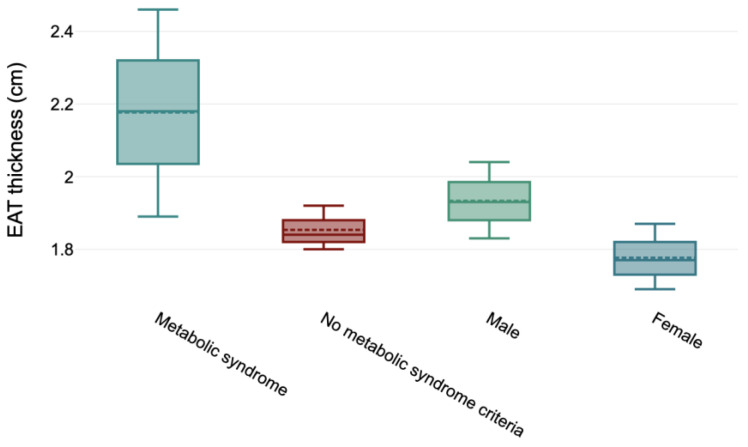
Association between epicardial adipose tissue (EAT) thickness values, sex, and metabolic syndrome according to 95 Intervale Confidence, median (line) and mean (dotted line).

Levels of EAT thickness established at the higher limit in children were linked to their being older (ρ = 0.11; *p* < 0.001), a higher BMI (ρ = 0.29; *p* < 0.001), WC (ρ = 0.28; *p* < 0.001), WHtR (ρ = 0.22; *p* < 0.001), SBP (ρ = 0.14; *p* < 0.01), DBP (ρ = 0.11; *p* < 0.05), the percentage of body fat (ρ = 0.24; *p* < 0.001), and lower HDL levels (ρ = −0.13; *p* < 0.01). Besides this, the EAT value was associated with being male (ρ = 0.14; *p* < 0.01) and presenting metabolic syndrome (ρ = 0.15; *p* < 0.01) (Figure 1).

The significance of EAT thickness was studied according to the children’s characteristics and cardiovascular factors through linear multiple regression, indicating significant links for height (*p* < 0.01), BMI percentile (*p* < 0.01), WC (*p* < 0.01), and WHtR (*p* < 0.01) (Table 2). The age (SD = 0.21; t = 1.679; 95.0% confidence interval −0.004 to 0.046, *p*-value = 0.095), levels of HDL (SD = 0.76; t = −0.76; 95.0% confidence interval −1.098 to 0.273, *p*-value = 0.27), or triglycerides (SD = 0.1; t = 1.12; 95.0% confidence interval 0.003 to 0.03, *p*-value = 0.092), cholesterol, or blood pressure were not linked (*p* > 0.05). Still, when these values were segmented differently between standard values and values above the intervals, EAT thickness was related to cardiovascular risk factors (Table 2). EAT thickness was associated with high blood pressure (*p* < 0.05), alterations in LDL (*p* < 0.05) [34], metabolic syndrome (*p* < 0.05), and being male, although high levels of HbA1c were not associated with EAT (*p* = 0.6). Moreover, in the case of overweight children, the EAT value was only linked to BMI (95% CI 1.048–1.129) (*p* > 0.01).

## 4. Discussion

The current research has shown that EAT levels in a rural pediatric population were initially associated with gender, BMI percentiles, blood pressure, WC, WHtR, cholesterol levels, triglycerides, and metabolic syndrome. Higher EAT levels for intervals estimated for age were ultimately linked to being of the male gender, BMI percentiles, WC, elevated blood pressure, higher LDL levels, and metabolic syndrome. Interestingly, despite previous analyses suggesting a possible link, HbA1c was not associated with elevated EAT levels in this study [5,35].

Previous research has shown that fat distribution and its quality in children are associated with subcutaneous abdominal fat and height, as reflected by WC, WHtR, and BMI [36,37,38,39]. Despite the results, the authors indicated that differences in EAT levels account for variations across ethnicity [37], age, gender, and geographic groups [38,39]. Santos et al. argued that WHtR is a superior index for overall adiposity compared to central adiposity and is a more accurate predictor of EAT than BMI [40]. This partly contradicts the model since the BMI had a higher association with EAT levels [37,41]. Chambers et al. [42] also noted that diabetes increases EAT values, although, in our research, HbA1c levels, pre-diabetes, and EAT were not linked, suggesting that prediabetes does not present in the form of higher levels of EAT thickness according to the age limit [32]. These differences could be due to the model, age limit, ethnic background, or differences between rural and urban areas.

EAT values have consistently been associated with variables such as age, gender, waist circumference, systolic BP, diastolic BP [42], hypertension, hypertriglyceridemia, and metabolic syndrome [41]. The final model indicated which variable influenced the EAT values, highlighting three cardiovascular factors (LDL values, being overweight, and higher BP) and metabolic syndrome. These associations matched prior studies in the older samples [20,42] and on the American continent [32].

Elevated EAT values have been linked to more than three or more cardiovascular risk factors [32] and are associated with an increased likelihood of cardiovascular diseases (CVD) in adulthood [38]. This makes the early detection of EAT alterations and the related risk factors, such as visceral fat and secondary atherosclerosis, critical for improving long-term health outcomes [36]. Several studies suggest that EAT thickness is linked to anthropometric factors such as BMI and glycemic profile, which are, in turn, associated with fat quality and quantity [37,38,39,40,41]. These results partially corroborate the relevance of gender [6,10], LDL levels [39], and metabolic syndrome regarding EAT levels [43].

Contrary to the findings of this study, which did not establish an association between higher EAT levels and HbA1c, research conducted on children with and without type 1 diabetes in Turkey indicated that EAT may serve as a specific and sensitive marker for insulin levels and resistance, making it highly relevant for diabetes management [5].

Finally, the results suggest that therapeutic interventions aimed at reducing obesity and BMI in adults have been shown to improve cardiovascular risk factors and decrease adipose tissue levels [1,37]. Applying similar interventions to children and adolescents could set an important precedent for the primary prevention of cardiovascular disease and promote healthier development into adulthood, particularly in underserved areas such as rural communities with limited access to healthcare [22].

The limitations of this study and its cross-sectional design are that it does not allow us to assess whether these associations are maintained until adulthood, so it would be interesting to propose follow-up or intervention studies and compare these results with those from urban areas and the socioeconomic factor. Another limitation, due to the voluntary nature of the study as it involved minors, was the reduced sample size compared with studies carried out in adults. The strengths of this study included the delimited population studied, and the fact that a wide range of factors was included, including the use of the echocardiogram technique to provide more precise data regarding EAT.

## 5. Conclusions

The current study aimed to describe EAT values in a Spanish pediatric population from a rural town and explore the associations between EAT and anthropometric measures, blood pressure, lipid and glucose profiles, and metabolic syndrome. The findings indicate that children with higher BMI who are in the overweight range in this rural area are significantly in the high range of EAT levels, with strong associations observed with WC and WHtR. Furthermore, increased levels of EAT at the higher limits for age groups were linked to three cardiovascular risk factors, including high blood pressure, elevated LDL levels, the presence of metabolic syndrome, and being male.

As a pioneering study in Spain, these results highlight the need for further research to understand better the relationships between EAT and cardiovascular risk factors in pediatric populations. Investigating these associations in rural and urban settings is essential to assess the long-term cardiovascular impact on children as they transition into adulthood.

## Figures and Tables

**Table 1:** Initial analysis of the characteristics of the sample.

	Total	Normal Weight (n = 114)	Overweight (n = 113)	*p*-Value
Age (years)	10.94 (2.86)	11.02 (3.13)	10.84 (2.59)	0.74
Height (cm)	144.07 (16.86)	142.39 (17.46)	145.33 (14.87)	0.14
Weight (kg)	44.06 (16.86)	36.91 (13.09)	50.98 (16.75)	<0.001
BMI (kg/m^2^)	20.29 (4.54)	17.42 (2.22)	23.35 (3.93)	<0.001
WC (cm)	66.03 (11.31)	59.50 (7.26)	72.69 (10.82)	<0.001
WHtR	0.45 (0.06)	0.41 (0.032)	0.49 (0.0538)	<0.001
BF%	24.76 (8.59)	18.54 (5.6)	31.12 (6.02)	<0.001
FF%	31.74 (4.34)	32.70 (5.06)	30.78 (3.24)	<0.01
EAT thickness (cm)	1.86 (0.53)	1.7 (0.43)	2.06 (0.61)	<0.001
SBP (mmHg)	110.40 (10.88)	107.67 (10.73)	112.85 (10.07)	<0.001
DBP (mmHg)	68.27 (6.52)	67.41 (5.99)	69.13 (6.92)	0.16
HbA1c	5.32 (0.29)	5.29 (0.27)	5.34 (0.32)	0.36
HDL cholesterol (mg/dL)	57.06 (12.81)	59.24 (12.38)	55.97 (12.94)	<0.01
LDL cholesterol (mg/dL)	94.63 (24.37)	89.12 (21.03)	100.25 (26.35)	<0.001
Total cholesterol (mg/dL)	167.34 (29.43)	162.58 (26.04)	172.18 (32.02)	<0.01
Triglycerides (mg/dL)	73.00 (39.79)	66.69 (30.42)	79.43 (46.92)	<0.05
Frequency	Total	Normal weight (n = 114)	Overweight (n = 113)	*p*-value
Being male	120 (53.1%)	59 (51.8%)	61 (54.0%)	0.89
Positive criteria for metabolic Syndrome	13 (5.7%)	1 (0.9%)	12 (10.6%)	<0.01

BMI: Body mass index; WC: waist circumference; WHtR: waist-to-height ratio; BF%: body fat percentage; FF%: free fat percentage; EAT: epicardial adipose tissue; SBP: systolic blood pressure; DBP; diastolic blood pressure; HbA1c: glycated hemoglobin.

**Table 2:** Adjusted model for epicardial adipose tissue (EAT) thickness and the associations with anthropometric and cardiovascular factors.

Anthropometric Variables	Standard Deviation (SD)	*t*-Test	95.0% Confidence Interval		*p*-Value
Height (cm)	0.014	−1.111	−0.061	−0.005	0.009
BMI percentile	0.065	0.339	0.075	0.329	0.002
WC (cm)	0.032	3.153	0.037	0.159	0.002
WHtR	4.546	−1.468	−22.818	−4.554	0.003
Cardiovascular Risk Factors	Standard Deviation (SD)	*t*-Test	95.0% Confidence Interval		*p*-Value
Being male	0.072	2.991	0.073	0.355	0.003
High blood pressure	0.145	2.283	0.045	0.617	0.033
High levels of LDL	0.272	−2.052	−0.31	−0.007	0.041
Metabolic Syndrome	0.158	2.074	0.060	0.675	0.019
High levels of HbA1c	0.107	0.107	−0.265	0.155	0.6

## Data Availability

Data is available and under consideration from the authors.
